# A Novel Approach to Assessing the Potential of Electronic Decision Support Systems to Improve the Quality of Antenatal Care in Nepal

**DOI:** 10.9745/GHSP-D-23-00370

**Published:** 2025-08-14

**Authors:** Biraj Man Karmacharya, Seema Das, Abha Shrestha, Abha Shrestha, Sulata Karki, Rajani Shakya, Emma Radovich, Loveday Penn-Kekana, Clara Calvert, Oona M.R. Campell, Ona L. McCarthy

**Affiliations:** a Department of Public Health and Community Programs, Kathmandu University School of Medical Sciences, Dhulikhel, Nepal.; b Research and Development Division, Dhulikhel Hospital Kathmandu University Hospital, Dhulikhel, Nepal.; c Department of Obstetrics and Gynecology, Kathmandu University School of Medical Sciences, Dhulikhel, Nepal.; d Department of Community Medicine, Kathmandu University School of Medical Sciences, Dhulikhel, Nepal.; e Faculty of Epidemiology and Population Health, London School of Hygiene & Tropical Medicine, London, United Kingdom.; f Usher Institute, University of Edinburgh, Edinburgh.

## Abstract

**Introduction:**

Electronic decision-support systems (EDSSs) aim to improve the quality of antenatal care (ANC) through adherence to evidence-based guidelines. We assessed the potential of the mHealth integrated model of hypertension, diabetes, and ANC EDSS and the World Health Organization EDSS to improve the quality of ANC in primary-level health care facilities in Nepal.

**Methods:**

From December 2021 to January 2023, we conducted a mixed-methods evaluation in 19 primary-level ANC facilities in Bagmati Province, Nepal. Implementation was from March 2022 to August 2022. We conducted a health facility survey, ANC clinical observations, longitudinal case studies and validation workshop, in-depth interviews, monitoring visits, research team debriefing meetings, health care provider attitude survey, and stakeholder engagement and feedback meetings. Results were integrated using concurrent triangulation to develop explanations about the EDSS implementation process and the effects observed.

**Results:**

We identified 9 themes on implementation challenges that hindered the EDSS from generating the desired improvements to ANC quality. Facility readiness and provider confidence in using the EDSS were mixed. It was not always used or used as intended, and the approach to ANC provision did not change. EDSS inflexibility did not reflect how staff made decisions about pregnant women’s needs or ensure that tests were done at the right time. There was mixed evidence that ANC staff believed that the EDSS benefited their work. The EDSS did not become fully integrated into existing health systems. Engagement of essential stakeholders fell short.

**Conclusion:**

Different understandings of and inconsistent use of the EDSS highlighted the need for increased training and support periods, greater stakeholder engagement, and further integration into existing health systems. Our novel approach to integrating findings from multiple substudies offers uniquely valuable insights into the many factors needed for the successful implementation of an EDSS to improve the quality of ANC in Nepal.

## Abstract (precis)

An electronic decision support system alone is not enough to provide quality antenatal care in the Nepalese setting.

## Teaser

**Key Findings:**

- Electronic decision support systems (EDSSs) are tools that can improve health care providers’ decision-making, promote adherence to evidence-based guidelines, and support clinical tasks.
- We assessed the effect of implementing 2 EDSSs in improving the quality of antenatal care (ANC) in select facilities in Nepal by improving provider’s adherence to ANC guidelines and ability to detect and manage higher-risk pregnancies.
- Although some changes in clinical provision were observed, some ANC components were not consistently provided during visits or at the right time.
- Possible reasons for the lack of improved quality of care include inadequate staff training, inflexibility of the EDSS design, contextual factors, and inadequate integration with existing health systems.

**Key Implication:**

- The findings of this study provide evidence to policymakers, program managers, and researchers on what factors must be considered for effective and sustainable implementation of EDSS in low-resource settings.

## INTRODUCTION

Nepal has made extraordinary progress in improving coverage of maternal health services, with more than 90% of women reported receiving antenatal care (ANC) from a skilled provider in 2022.^1^ Of the 79% of live births delivered in a health facility, 62% of these facilities were in the public sector.^1^ Home births are around 19% of live births nationally, down from 91% in 1996, with variation by province.^1^ As the coverage of ANC has increased, improving quality of care has become a growing strategic priority for the government.^2^^,^^3^

World Health Organization (WHO) guidance states that all pregnant women should receive essential services throughout pregnancy, including regular ANC visits, preventive interventions, and screening for potential complications.^4^ Electronic decision support systems (EDSSs) are clinical decision-support tools that integrate clinical and demographic client data with clinical practice guidelines.^5^^–^^9^ EDSSs aid health care providers’ decision-making and promote adherence to evidence-based practices through alerts, checklists, or information provided at the point of care.^10^^,^^11^ EDSSs can support clinical tasks, such as client monitoring, drug prescribing, and formulating diagnoses or treatment suggestions.^10^ They can be particularly relevant in lower-level health facilities that lack senior or specialist health care providers.^12^ However, evidence for the effectiveness of EDSSs on quality of care and health care provider performance remains mixed.^10^^,^^11^^,^^13^^–^^15^

We report on a novel approach that integrated the results of a variety of mixed-methods studies to assess the potential of 2 EDSSs designed for use by health care workers to improve the quality of ANC in Nepal: the mHealth integrated model of hypertension, diabetes, and antenatal care (mIRA EDSS) and the WHO digital ANC module (WHO EDSS).^16^ A cluster-randomized control trial is ongoing in India to evaluate the effectiveness of the mIRA EDSS.^17^ Throughout this article, when we refer to the EDSS, we are commenting on both the mIRA EDSS and the WHO EDSS together.

We assessed the potential of 2 EDSSs to improve the quality of ANC in select facilities in rural Nepal.

The specific objectives of this study were to (1) assess the effect of the EDSS on the quality of ANC and implementation outcomes, including acceptability, adoption, appropriateness, feasibility, and fidelity; (2) document and understand the implementation process and how the EDSS became part of existing systems; (3) explore how contextual factors impacted implementation and effectiveness of the EDSS; and (4) investigate the mechanisms through which the EDSS changed the delivery and quality of ANC.

## METHODS

### Study Design

This was a theory-driven, mixed-methods study. The study included 8 substudies: a health facility survey, ANC clinical observations, longitudinal case studies and validation workshop, in-depth interviews, monitoring visits, research team debriefing meetings, health care provider attitude survey, and stakeholder engagement and feedback meetings. This study focused on overall acceptability, adoption, appropriateness, and feasibility of both EDSSs in Nepal. Thematic grouping was used to organize the shared findings and address the positive aspects and challenges encountered during EDSS implementation.

### Setting

We worked in 4 predominantly rural districts in Bagmati Province, Nepal: Kavrepalanchok, Sindhupalchowk, Sindhuli, and Dolakha. Twenty primary-level ANC facilities were matched based on facility type and ANC client volume and randomly allocated to receive the mIRA EDSS (n=10) or the WHO EDSS (n=10). After allocation, we dropped 1 facility (randomized to WHO EDSS) because it had few ANC clients. The remaining sites were government health posts (n=12), primary health care centers (PHCs, n=4), and Dhulikhel Hospital outreach centers (n=3).

### Interventions

**FIGURE 1:**
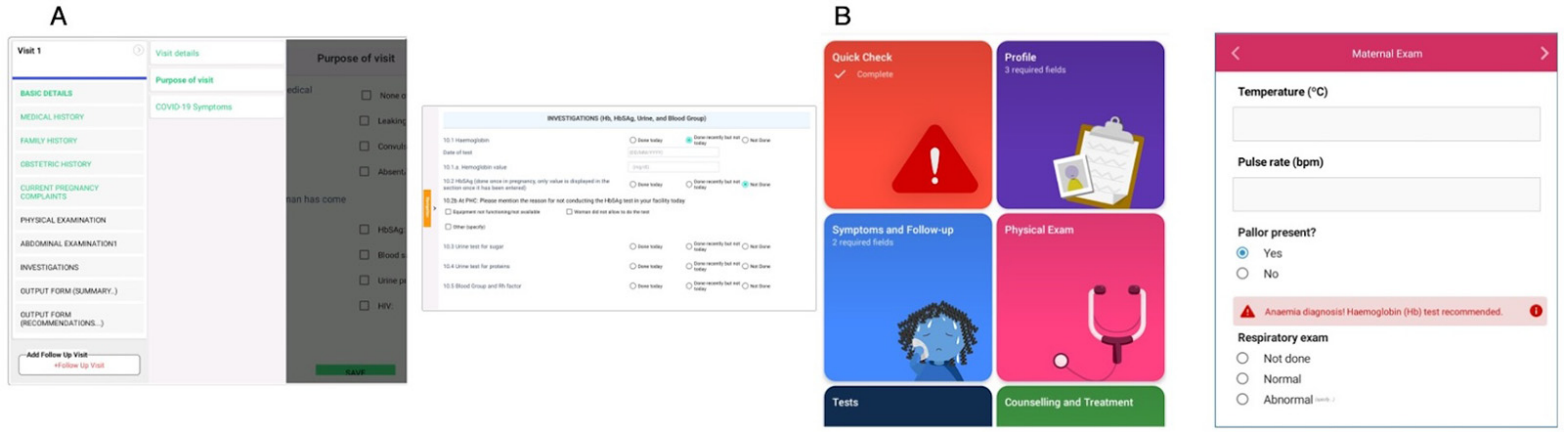
Screenshots of Two EDSSs Implemented in Nepal ANC. A: mHealth Integrated Model of Hypertension, Diabetes, and ANC EDSS; B: WHO Digital ANC Module Abbreviations: ANC, antenatal care; EDSS, electronic decision support system; WHO, World Health Organization.

The EDSS intervention was implemented from March 2022 to August 2022 and included 2 broadly similar EDSSs that aim to improve providers’ adherence to routine ANC guidelines and detection and management of higher-risk pregnancies. The mIRA EDSS was developed by the Public Health Foundation of India, Dhulikhel Hospital, Kathmandu University Hospital, and the London School of Hygiene & Tropical Medicine for use in India and Nepal. Intended to be used by providers during each ANC consultation, the mIRA EDSS includes prompts aligned to routine ANC guidelines, bespoke diagnosis, and treatment content and pop-up prompts for gestational diabetes, pregnancy-related hypertension, and anemia. The WHO EDSS facilitates the adoption of WHO ANC guidelines,^4^ focusing on routine care, with checklists for screening and referral but not on the treatment of pregnancy complications. It incorporates a “facility infrastructure parameter” (e.g., whether ultrasound is available).^16^ Supplement 1 provides a description of the EDSS intervention (Figure 1), and Supplement 2 is the checklist for reporting mHealth interventions.

### Staff Training and Tablet Provision

Each health care facility was given a functional tablet with an EDSS and a SIM card for cellular Internet to allow data inputted in the EDSS to sync to servers during power outages. The local municipality selected 1 auxiliary nurse midwife (ANM) per facility to attend an in-person 3-day training workshop in March 2022 on using either the mIRA or WHO EDSS. ANMs were subsequently supported to use their allocated EDSS by an onsite research team for 1 month and provided with continued technical support and monitoring visits for the next 6 months.

### Theory of Change

**FIGURE 2:**
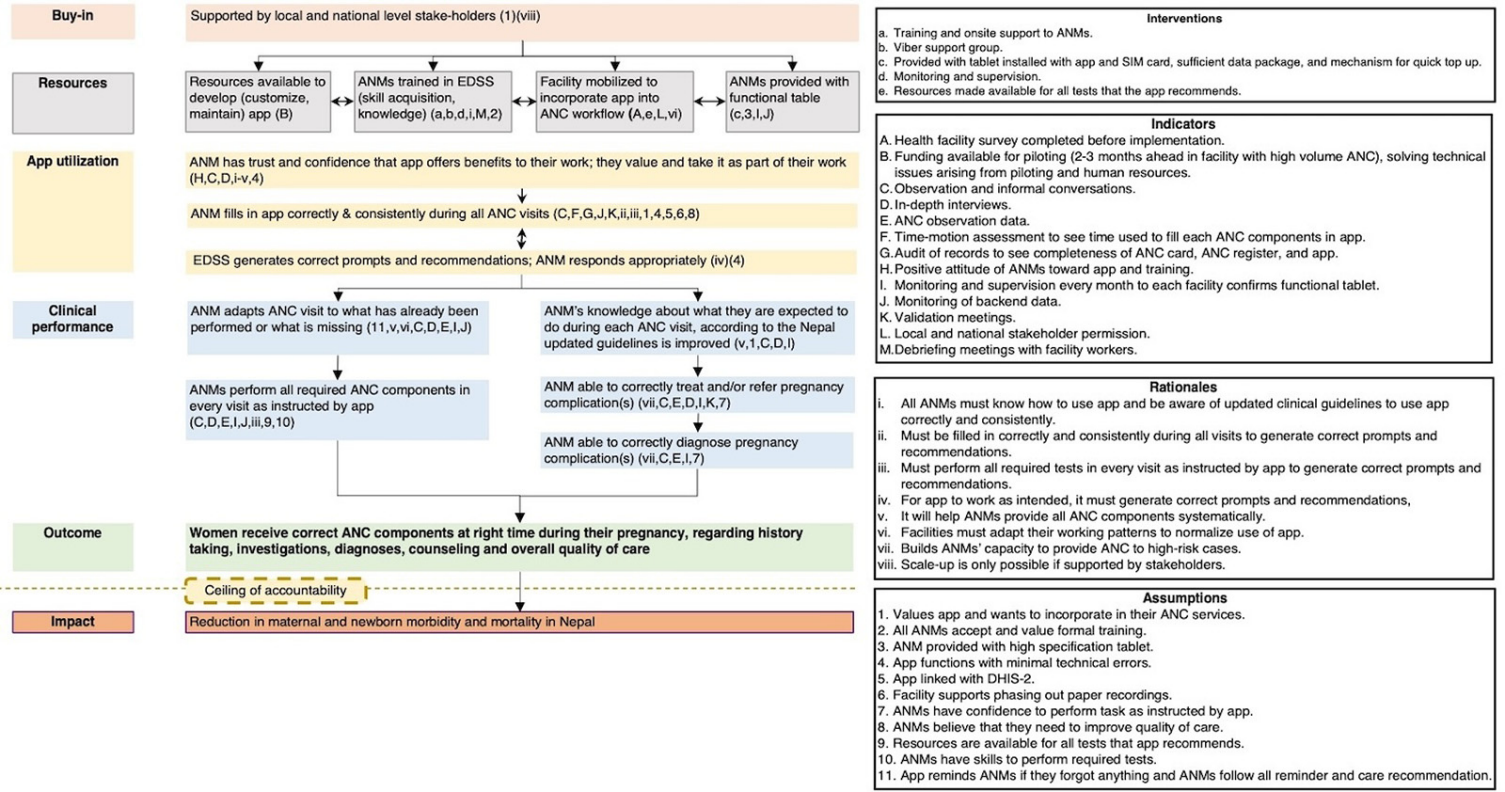
Theory of Change for Effect of Electronic Decision Support System Intervention on Provision of Antenatal Care, Nepal

A conceptual framework was developed based on how we thought the intervention would improve adherence to ANC guidelines and enhance detection and management of pregnancy complications. After EDSS implementation, the framework evolved into a theory of change (Figure 2 ^18^) and was refined during the course of the intervention.

### Data Collection and Analysis

The following data collection methods were used (methodological details are available elsewhere^19^).

#### Health Facilities Survey

Before the EDSS was implemented in the 19 facilities, we interviewed the facility in-charges and ANMs and collected data on the availability and functioning of equipment and medicines. We calculated the percentage of facilities with key equipment and medicines available overall and stratified by facility type.

#### Clinical Observations

We trained the research team to observe at least 30 ANC visits in each site before and at least 30 after the EDSS was implemented. An ANC quality score was calculated by adding 1 point for each of the following ANC components provided by the health care worker during consultation: measurement and recording of blood pressure, blood glucose test, urinary dipstick test, and hemoglobin test (maximum score of 4). We compared mean ANC scores among observations before and after implementation.

#### Longitudinal Case Studies and Validation Workshop

Two researchers (SD and SK) conducted 3 visits (lasting up to 2 weeks) in each of 4 purposively chosen facilities (2 health posts and 2 PHCs). One visit was conducted before implementation and 2 were during, totaling about 2 months of observation in the PHCs and about 1 month in health posts. We subsequently conducted a validation workshop in each facility to review findings with the ANMs and facility in-charges. All the notes from observations and informal conversations with facility staff and pregnant women and validation workshop notes were analyzed using a thematic approach.

#### In-Depth Interviews

At the end of longitudinal case studies, we interviewed all ANC staff (N=16); data were analyzed using a thematic approach.

#### Monitoring Visits

Trained researchers conducted 1 monitoring visit per month for 4 months after EDSS implementation. Quantitative data were analyzed descriptively, and notes were analyzed using a thematic approach. EDSS usage data were generated through use of the EDSS and stored on the server after syncing. These backend data were extracted after implementation and analyzed descriptively.

#### Research Team Debriefing Meetings

The research team participated in weekly debriefings (N=5) while providing onsite support. One meeting was held separately for mIRA and WHO EDSS intervention sites, and the remaining 3 were combined. Debriefing notes were analyzed thematically.

#### Health Care Provider Attitude Survey

At the end of onsite support, a self-administered health care provider attitude survey was completed by all ANC staff in each intervention site (N=43). Data were analyzed descriptively.

#### Stakeholder Engagement and Feedback Meetings

Four meetings were held periodically with local (N=35) and national (N=20) to discuss the intervention’s progress and obtain feedback. National stakeholders had expertise in maternal health, health informatics, and noncommunicable diseases. Local stakeholders included municipal health coordinators, facility in-charges, female community health volunteers, and ANMs. All the meeting notes were analyzed thematically.

### Data Analysis

Supplement 3 summarizes the studies and methods. These were given equal weight in the analysis using concurrent convergent triangulation.^20^^,^^21^ Quantitative and qualitative data analyses were initially done separately. Then, similar content areas in the different datasets were compared, contrasted, and synthesized. Where findings diverged, the research team discussed and reexamined results, reflecting on the quality of data and strengths/weaknesses of the different studies to arrive at the most valid type of interpretation.

### Ethical Approval

We received ethical approval from the Kathmandu University School of Medical Sciences (IRC, KUSMS 25/22), Nepal Health Research Council (ref: 2695), and London School of Hygiene & Tropical Medicine (ref: 25094-1). All municipalities with intervention facilities also gave approval. We obtained written informed consent from all health facilities and ANC staff. Interviews with ANC staff and validation meetings were audio-recorded with prior consent from ANC staff and facility in-charges. Additionally, verbal informed consent from health care providers involved in ANC (e.g., medical doctors and laboratory personnel) and from pregnant women was obtained before conducting informal conversations. Further, verbal informed consent was obtained before stakeholder engagement and feedback meetings for using information for research purposes. To maintain confidentiality, all the transcripts and notes were anonymized, and data were de-identified. We also obtained written informed consent from pregnant women before extracting data from their ANC cards, taking pictures of their ANC cards, or observing their ANC visits.

## RESULTS

Results are reported in relation to the first 4 domains of the theory of change: buy-in, resources, app utilization, and clinical performance (Figure 1) and organized around the 9 key messages triangulated by findings from the multiple data collection methods.

### Buy-In

#### Engagement of Essential Stakeholders Fell Short

In general, all the stakeholders were interested in the EDSS evaluation, valued our novel approach, and considered digital health as a government priority area.

All the stakeholders were interested in the EDSS evaluation, valued our novel approach, and considered digital health as a government priority area.

*Government is also considering digitalization in health and therefore you can use Government Integrated Data Centre of web hosting which helps data storage for long term. It is necessary to provide complete services for ANC, delivery and postnatal care.* —Integrated health information management system chief, final national stakeholders meeting

ANC staff attending stakeholder meetings expressed appreciation for EDSS features, including reminders, organized consultation, and minimal writing. At the final local stakeholders meeting, a few ANC staff recommended implementing this type of EDSS at the national level, digitizing ANC registers, and then integrating it into Nepal’s DHIS2. The self-administered health care provider survey conducted partway through implementation showed that 98% of ANC staff saw the potential value of EDSS, and about 95% of ANC staff believed that using an EDSS was worthwhile.

Although stakeholders were generally positive during meetings, they did not actively engage in the implementation or provide dedicated support to implement EDSS in the facilities. Stakeholders were also concerned about the data security and privacy.

### Resources

#### Mixed Levels of Facility Readiness to Implement Recommendations

**FIGURE 3:**
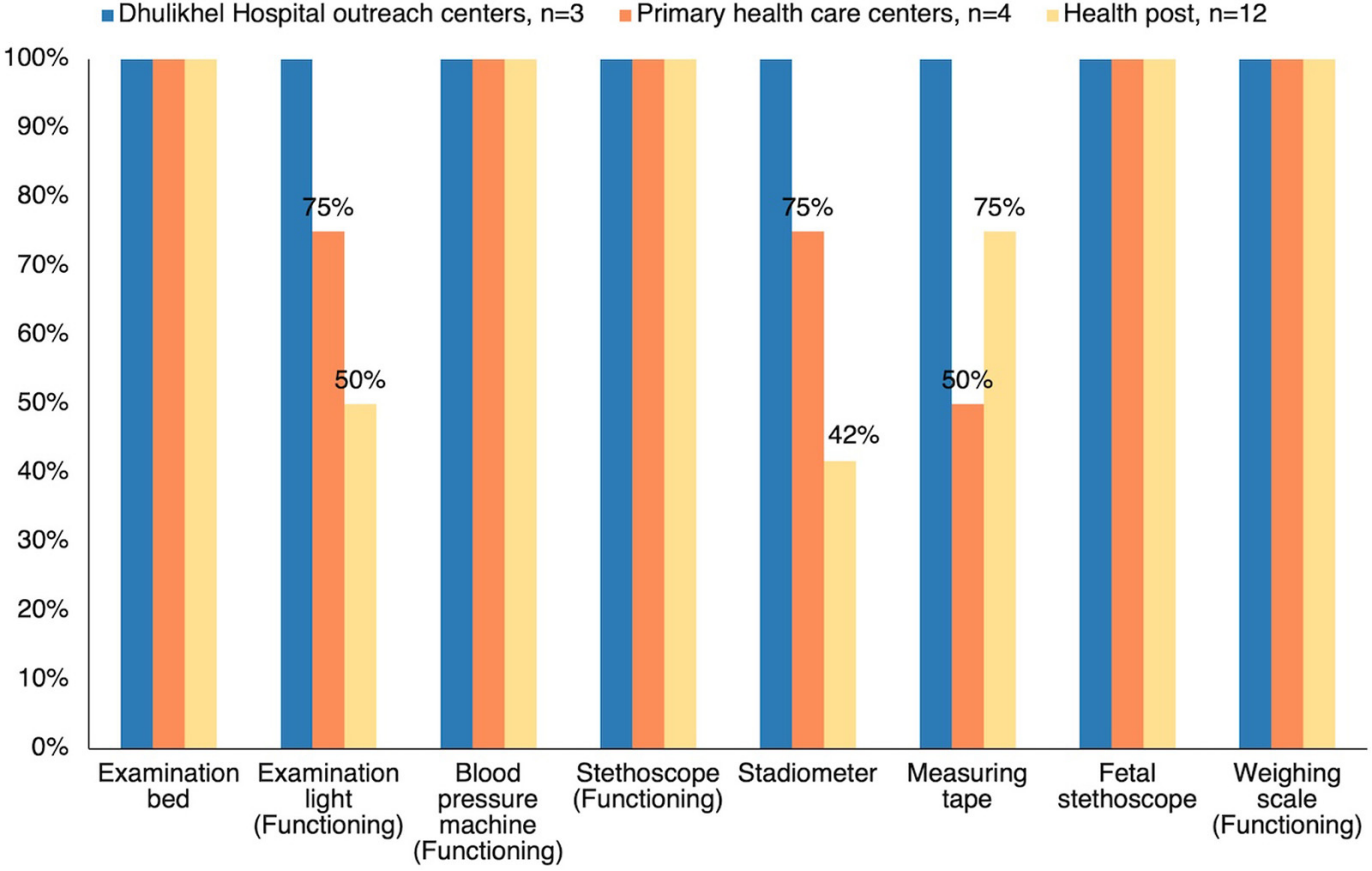
Availability of Basic Antenatal Care Equipment at Intervention Facilities, Nepal

The health facility survey confirmed that all facilities had access to electricity but that 25% of health posts lacked Internet connectivity, which we redressed by providing SIM cards. Most facilities had the 8 key equipment components (Figure 3), but some were missing the urine protein test (41%), the hemoglobin test (23%), or the glucose test (21%). When we introduced the EDSS, we provided all facilities with a glucometer, glucose strips, and 75-gram glucose, as this was not a standard component of ANC provided by the government. No EDSS tablets were lost or broken during the intervention, although 1 charging cord was replaced.

#### Training Staff Did Not Necessarily Lead to Confidence in Use

Some staff found the EDSS slightly difficult to use at first, but most formally trained staff were able to use it and were confident they would improve with continued use.

*ANM said in the beginning, it takes a lot of time for us to fill in. Due to continuous filling in the app, my eye hurts. We have to fill in the questions by spending a lot of time, now it is a little easier for me personally. When asking about history, I can ask without looking one by one, and I don’t have to wait for the advice to be given by the app, I do not need to read. Now it has become easy.* —ANM, longitudinal study notes

During the longitudinal study, we observed that many staff who were not formally trained to use the EDSS were not interested in using it. During in-depth interviews, some conveyed that they needed formal training to feel a sense of responsibility.

*After training, they feel like it is their responsibility and might think of it as their own work as well. I feel like other staff think it is only the responsibility of staff who took the formal training.* — ANM, in-depth interview

Others found the onsite support beneficial and learned to use the EDSS, as noted during the longitudinal study. A few ANC staff said that they would be more conscientious in using the EDSS if incentives were provided. A small financial incentive was provided at 1 health post at the request of the facility in-charge and ANC staff; however, we did not observe greater engagement with the EDSS at this facility during the longitudinal study.

Factors, such as low ANC volume (<40 ANC consultations per year in most health posts), staff on leave, and a local election held during the onsite support phase that required 1–2 ANC staff from each facility to work for the elections, impacted the EDSS training. In facilities where ANC volume was low, staff said there were not enough opportunities to use the EDSS during onsite support.

*Trained ANC staff were on leave, and since then EDSS was not used. We haven’t worked on the tablet. We didn’t get an opportunity to use the EDSS due to low ANC flow.* — ANM, monitoring visits

High staff turnover affected at least 1 facility and impacted the readiness of the health facility to use the EDSS, but efforts were made to ensure that an EDSS-trained staff member was present.

### Electronic Decision Support System Utilization

#### Staff Did Not Always Use the Electronic Decision Support System and Did Not Always Use It As Intended

Backend data showed that the mIRA EDSS was used with only 37% of ANC clients (29 EDSS entries of 79 ANC register entries for the same dates) and the WHO EDSS in 81% (43/53) of clients. The monitoring visits, the longitudinal study, and the research team debriefings confirmed that ANMs did not always use the EDSS at every visit and in the presence of pregnant women. Instead, the ANMs photographed the ANC cards or wrote notes, which they would later enter into the EDSS after women left the facility at the end of the day or on another day.

Data showed that the mIRA EDSS was used with only 37% of ANC clients and the WHO EDSS in 81% of clients.

*They clicked the picture of an ANC card during busy hours and filled the data into EDSS when they were free.* —ANM, monitoring visit notes

Sometimes, ANMs copied the information from the ANC card when a woman left the facility for a laboratory test or copied information from the ANC register.

*They write the notes in a separate paper of medical history, family history, obstetrics history, investigations, and chief complaints and enter data later in EDSS after working hours during free time.* —ANM, monitoring visit notes

The monitoring visits and longitudinal study revealed that some ANC staff felt that the EDSS was too time-consuming to use in the presence of women. The EDSS was more likely to be completed during the consultation when there were 2 or more ANC staff present, with 1 staff member doing the examination and the other filling in the EDSS.

*First, they fill up the register and then examine the patient. One of us examines the patient and then we fill in the tablet. If we are alone, we examine the patient first, finish up everything and use the tab.* —ANM, in-depth interview

#### Mixed Evidence That Staff Believed That the Electronic Decision Support System Benefits Their Work

During in-depth interviews, ANC staff emphasized that the EDSS reminded them to organize the care through a checklist for history-taking, husband details, danger signs, and pregnancy complaints. All ANC staff expressed that they were guided by the EDSS to provide a systematic ANC consultation, and some ANC staff also expressed that the EDSS had guided them to perform more counseling.

*One thing is that it has helped us to go step-by-step through the ANC consultation process. —*ANM, in-depth interview

*I think this has guided me do more counseling.* —ANM, validation workshop

During the monitoring visits and longitudinal study, ANC staff identified problems with the EDSS, which meant that we had to update the software frequently. It became clear after implementation that the WHO EDSS required a higher specification tablet than those used in the facilities. Technical issues encountered with the EDSS included pages “hanging” and responding slowly, and, specifically for the WHO EDSS, miscalculating Nepali dates. These discrepancies, though ultimately resolved, created frustration and eroded the ANM’s trust in the EDSS.

*ANM said that the app is slow when we entered the investigation details in it and therefore time consuming. Also, it freezes and stops responding.* —ANM, longitudinal study notes

#### Inadequate Integration With Existing Health Systems Impaired Utilization

The organization of ANC hindered adequate utilization of the EDSS and its potential to improve clinical performance. The health facility survey showed that most of the health posts lacked laboratory and ultrasound services, so women were referred to PHCs or higher centers to perform tests and screening, which interrupted the flow of the visits. At the same time, women attending the PHCs only for tests or screening did not receive full ANC consultations and were not recorded in the EDSS, as noted in the longitudinal study.

Most ANC staff prioritized paper-based ANC records systems over the EDSS, explaining that the EDSS was for a short-term research intervention and was not integrated with monthly reporting systems. Paper-based records were seen as long-term, government work and, therefore, more important to complete. The EDSS was not linked to DHIS2, and if it had been, ANC staff felt that it would have decreased their reporting workload and increased EDSS use.

*If it can be made a government-owned program it will be continued and integration of software with DHIS-2 will be helpful for reporting.* —Medical doctor, longitudinal study notes

### Clinical Performance

#### Electronic Decision Support System Use During Consultations Did Not Guarantee That Tests Were Done at the Right Time

The EDSS was intended to prompt ANC staff to perform tests for pregnant women as required per trimester or at each ANC visit. Observations of ANC before and after implementation suggested the mean number of the 4 primary ANC components (measurement and recording of blood pressure and performance of blood glucose, urinary dipstick, and hemoglobin tests) increased (1.11 before and 1.56 after implementation, *P*=.09), but this could have been due to chance (Supplement 4). Compared to before implementation, performance of the urinary dipstick test (7.9% to 23.5%, *P*=.07), hemoglobin test (7.9% to 17.7%, *P*=.21), and blood glucose test (2.6% to 20.6%, *P*=.02) improved after EDSS implementation.

The EDSS prompted ANC staff to do more frequent tests as per the Nepali and WHO guidelines, but these were not part of their usual practice. Evidence from the longitudinal case study, in-depth interviews, monitoring visits, and validation workshops showed that ANMs did tests during the first ANC visit but did not repeat them unless they observed abnormal results or were advised by doctors to do so. Newer ANC staff (who might have been trained in newer guidelines) followed the practices of older staff in the facility. ANC staff also explained they limited tests out of concern for women’s financial conditions, as women were required to pay a small amount for tests in some facilities.

Evidence showed that ANMs did tests during the first ANC visit but did not repeat them unless they observed abnormal results or were advised to do so.

*No protocol for performing the test once. It was practiced for so long, in this facility, and might be same in the facilities with the similar settings. Also, women don’t have enough money to do tests frequently so if normal values of test results, then tests are not repeated. Sometimes, we also make decision based on clinical judgment, such as checking for blood pressure, edema, signs and symptoms of other risk conditions.* —Facility in-charge, validation workshop

In the longitudinal study, we observed 5 instances where ANC staff had not performed a test in a previous visit, and the EDSS reminder led them to perform the test. Also, ANC staff were trained to administer oral glucose tolerance tests to detect gestational diabetes using test kits we provided. Only 1 oral glucose tolerance test was logged during the ANC observations, although the longitudinal study and monitoring visits showed that ANC staff initiated and performed the test for most clients.

Among ANC observations where clients reported experiencing vomiting, vaginal bleeding, severe headache, decreased or absent fetal movement, severe abdominal pain, or blurred vision, the percentage of providers who took the appropriate action in response improved from 0% (0/7) before EDSS implementation to 100% (5/5) after (small numbers because only women reporting symptoms were included from among 72 ANC observations). However, the mean number of these symptoms (plus nausea) discussed decreased from 3.08 pre-implementation to 1.76 post-implementation (*P*<.01) (Supplement 4).

*Since we have explained danger signs during the first entry, so we don’t need to do it again in every visit and therefore it was marked as no symptoms observed in the section of EDSS.* — ANM, in-depth interview

#### Staff Did Not Substantially Change Approach to Antenatal Care Provision

We observed some examples of changes in provision. The ANC observations showed that even though there was space in the ANC card to record height, it was not measured or recorded before EDSS implementation. After implementation, ANC staff started measuring and recording height in the EDSS and on ANC cards.

However, not all required ANC components (as instructed by EDSS) were performed at every visit. It was noted in the longitudinal study that ANMs referred clients to a doctor rather than follow the EDSS recommendation and that most ANC staff focused on providing basic ANC. The longitudinal study, monitoring visits, and validation workshops all showed staff tended to refer more complicated ANC consultations either to medical doctors of the facilities or to the nearby higher-level center. The health facilities survey showed that all intervention PHCs, Dhulikhel Hospital outreach centers, and 2 health posts had at least 1 doctor, and most of these facilities were close to higher-level centers that were easily accessible to pregnant women. In general, observations and interviews suggested that most of the ANC staff were satisfied with their own ANC practice. Most staff seemed to have adequate training and knowledge to provide ANC for low-risk women; however, they were always eager to acquire new skills and knowledge.

Not all required ANCe components were performed at every visit.

*I am satisfied with my practice. I think we are following the government’s guidelines. The government has told us to give medicines, and we have been following its instructions. We are doing the investigation that has been decided, and that is what we are practicing. So, I think it is sufficient while providing ANC because the guidelines given by the government are followed accordingly.* — ANM, in-depth interview

The times and days that ANC was provided also affected opportunities to change behavior. In all facilities, ANC flow was high between 10 am–1 pm. During this time, ANC staff felt pressure to provide ANC quickly and found it difficult to use the EDSS. The health facility survey showed that 7 health facilities had a designated ANC day, and only 1 of them had ANC days twice a week. While ANC consultations were provided on non-ANC days, pregnant women were generally asked to attend on ANC days. On these ANC days, ANC flow was high.

*The EDSS required more time than usual, if we do it from the start to counseling, it takes 1 hour for a patient. And then, ANC clients become impatient. Therefore, ANC was organized as per ANC clients’ demand.* —ANM, in-depth interview

During a validation workshop, an in-charge mentioned that reorganizing or adding ANC days was difficult because they had different tasks for each day of the week, and this would impact other services provided at the facility on other days. A few ANC staff members also said that using the EDSS reduced their interaction with pregnant women.

#### Inflexibility of Electronic Decision Support System Design Did Not Reflect How Staff Made Decisions About Pregnant Women’s Needs

The EDSS was designed to provide all women with the same information at all visits, and other content was standardized as per the national protocol and WHO guidelines. However, ANC staff adapted consultations to be more personalized to pregnant women’s specific conditions and their needs at the time of consultation, considering their family and economic conditions. Most ANMs felt that counseling and information about danger signs were only needed once unless women complained about the problems.

The ANC observation data indicated that the mean number of the selected danger signs (severe vomiting, vaginal bleeding, severe headache, decreased or absent fetal movement, severe abdominal pain, or blurred vision) for which the provider told the woman she should return for help decreased from 2.66 (before implementation) to 1.56 (after implementation) (*P*=.01). Monitoring and longitudinal visits showed that many of the ANC staff marked counseling sections as complete in the EDSS rather than actually providing counseling to pregnant women as suggested by the EDSS.

ANC staff stated that they believed they provided care that met women’s needs. If women looked fine and had no complaints and they considered women’s personal issues (e.g., transportation, family issues/household chores, weather, and economic conditions), staff provided a quick ANC visit. If women reported any problems, then staff said they provided counseling accordingly and immediate referral. There was a small decrease in the mean number of 20 counseling-related components from before (8.82) to after (6.94) EDSS implementation (*P*=.04) (ANC observations, Supplement 4). In the in-depth interviews, ANC staff said they worried that if counseling was delivered while referring to the EDSS, pregnant women might think that they did not have enough expertise.

## DISCUSSION

Guided by our theory of change and mixed-methods approach, we identified 9 themes that deepened our understanding of how the EDSS was used and highlighted the complexities and challenges that prevented the EDSS from bringing the desired ANC quality improvements. We found that although stakeholders expressed interest in the EDSS and its features, they were not sufficiently engaged in the implementation. Some facilities lacked key components to perform tests, indicating different levels of readiness to implement the EDSS recommendations. Staff who were trained to use the EDSS did not feel confident using it at first and did not use the EDSS at every visit or as intended. There was mixed evidence that ANC staff believed that the EDSS benefited their work and evidence of inadequate integration of the EDSS with existing health systems. Use of the EDSS during the consultation increased the performance of some tests but did not guarantee that tests were done at the right time. Although there were some changes observed in ANC provision, staff did not perform all required components at every visit. The inflexibility of the EDSS design did not reflect how ANC staff made decisions about pregnant women’s needs.

### Potential Explanations for Lack of an Observed Effect

#### Lack of Adequate Training

The different understanding of the EDSS and how it is intended to be used highlights the need for broader training and increased support periods. Health care providers were using this EDSS for the first time; a 3-day training for 1 person in each facility with 1 month of onsite support appeared to be insufficient to generate a sense of responsibility among staff to use the EDSS and an appreciation of its potential. The literature suggests that change is more likely with longer trainings^22^^,^^23^ and implementation periods,^24^^,^^25^ but time constraints meant our intervention was implemented for a short period (6 months). Failing to formally train all staff can limit accountability.^8^^,^^26^ Performance-based incentives have been shown to increase job satisfaction and improve practices,^27^ within direct incentives, such as encouragement, recognition, and support, being highly desired and valued by staff.^8^ The effectiveness of financial incentives as a mechanism to promote behavior change has been mixed^28^; we did not observe meaningful changes in 1 facility where all staff were provided with a small incentive.

A 3-day training for 1 person in each facility with 1 month of onsite support appeared to be insufficient to generate a sense of responsibility among staff to use the EDSS and an appreciation of its potential.

#### Mismatch in Intended Benefits Versus Perceived Benefits

ANC staff held mixed views about the benefits of EDSS to their work despite expressing positive views toward the EDSS in initial interviews and surveys. The perceived benefits (guidance in organizing care through pop-ups and reminders and detailed history-taking and counseling section) may have been negated by the increased workload generated by dual documentation (the EDSS and paper-based records). This finding aligns with digital health studies conducted in African countries.^25^^,^^29^ In our intervention, it was not possible to completely replace the paper ANC records for the short intervention period. Nor did the EDSS aid monthly indicator reporting; a link to routine data monitoring might have made the EDSS more appealing to staff as it would potentially decrease their workload. Future development of the EDSS must satisfy these needs and expectations of staff to increase the potential for its success.^30^

#### Lack of Staff Awareness Regarding Current Antenatal Care Quality Guidelines

In general, ANC staff believed that they were providing quality ANC even before the EDSS implementation, which could explain why we did not observe a change in their approach to ANC provision. Our intervention was conducted during a transition period, where the official guidelines were moving from 4 to 8 recommended ANC visits. This shift had not been effectively communicated in all facilities, and staff did not always think that the 8 visits they were asked to do through the EDSS were practical for women. ANC staff believed that they were providing client-centered care regarding contextual factors (ANC client needs and their social and economic condition) and organizational factors (workload, staff turnover, health system, and supervision). This included providing counseling based on perceived need and mostly during the first visit, with birth preparedness and emergency readiness during the last visits. A similar pattern was confirmed in ANC observations in a national health facility survey.^31^ It is possible that the combination of minimal communication regarding the new guidelines to staff and belief among staff that counseling is most important at the first visit contributed to the lack of engagement with the EDSS counseling prompts.

During ANC consultations, we observed that the EDSS had a positive influence on history-taking practices, which is a similar finding to a study conducted in Ghana.^24^ We also found slight improvements in the performance of screening tests. In contrast, contextual difficulties with digital decision support systems have been shown to surpass their perceived usefulness,^8^ with similar findings reflected in our intervention.

#### Inflexibility of the Design

The EDSS design did not reflect how ANC staff made personalized decisions about pregnant women’s needs. This inflexibility affected how staff used the EDSS, making it difficult to facilitate the desired change in ANC staff behavior. The EDSS’ emphasis on standardization made it less of a decision-support tool and more of a guideline-adherence tool. Personalized care should allow ANC staff to make informed decisions considering each woman’s needs at all visits. However, standardization guarantees that all women receive the same information for each visit. It takes more time to understand the individual client’s circumstances and needs. Future EDSS development could take adaptability and personalization into account.

The EDSS design did not reflect how ANC staff made personalized decisions about pregnant women’s needs.

#### Contextual Challenges

Several contextual factors that we were unable to fully account for in the evaluation design may have impacted implementation. The COVID-19 pandemic and software development delays meant the training and implementation period was shorter than originally planned. Additionally, unresolved software issues may have affected the usability and functionality of the EDSS. Changes in guidelines from 4 to 8 ANC visits and municipality political leadership, upgrading of facilities, and technical difficulties may also have disrupted use of the EDSS.

#### Inadequate Integration With Existing Health Systems

Finally, inadequate integration of EDSS with existing health systems impaired its utilization. The EDSS recommended more tests than what was done in usual practice. Despite assumptions and official policy that all ANC is provided free, some tests were not free, which meant some women might not be able to afford repeat tests suggested by EDSS. Having adequate supplies and considering service fees are fundamental to realizing the benefits of EDSS and normalizing its use in daily work.^25^ Our small-scale intervention did not link across different health facilities, which could have contributed to greater use of the EDSS and more continuity of care because pregnant women attended different facilities during the antenatal period. Active participation of the government is essential in considering the needs and priorities of the health system and integration of national strategies to increase the sense of acceptance and recognition among the ANC staff.^6^ A study reported that strong commitment and support from the government is essential for promoting sustainability and scale-up.^32^

### Strengths

This is the first time this type of digital intervention has been implemented in ANC in Nepal. We demonstrated that with training, ANMs have the technical skills to use mobile digital technology to support consultations. Our novel approach allowed us to triangulate qualitative and quantitative data and provided an in-depth appreciation of the multitude of contextual factors influencing EDSS implementation. The longitudinal study, in particular, offered unique and valuable insights into how ANC staff perceived the EDSS and incorporated it into their workflow and why.

### Limitations

Implementation and evaluation of the EDSS were conducted in a small number of facilities, without a comparison group, limiting the conclusions we could draw from the findings. The small sample size for some studies, notably the ANC observations, limited further analysis and stratification (e.g., comparisons between mIRA and WHO EDSS facilities or adherence to guidelines in first versus follow-up ANC visits). Further, development of the intervention took place during the COVID-19 pandemic, which limited opportunities to engage users in the design that could have addressed some of the implementation challenges.

## CONCLUSIONS

There are indications that the EDSS can improve some aspects of quality of care; however, we demonstrate that the EDSS alone is not enough to improve the quality of ANC provided. Health workers’ responses to interventions are complex.^33^ This intervention provides evidence on the implementation considerations that can inform policy and future research on digital health interventions. Future EDSS development and implementation could maximize EDSS potential by considering the financial implications on the care receiver, health system resource allocation, staff workload and motivation, alignment with the needs of staff and clients, intensive training on EDSS use and content to all ANC staff, and continuous support and supervision from the government. An EDSS will be limited in improving the quality of ANC in settings where multiple barriers exist to providing recommended care. However, greater involvement of all stakeholders, including ANC staff, pregnant women, and policymakers, in designing and implementing an EDSS intervention may help to address some of the challenges encountered in this evaluation.

Before scale-up can be considered, the findings of this evaluation must be used to optimize the EDSS. These efforts could include improving staff training, redesigning the EDSS to be more flexible, and improving EDSS integration with existing systems. Once this optimization is complete, further research will be required to evaluate the effectiveness, financial implications, and sustainability of the EDSS in the Nepali context. This intervention demonstrated the benefit of using multiple methods in understanding implementation successes and failures.

## Notes

**Peer Reviewed**

**First Published Online:** January 9, 2025.
